# Supplementary material for: Identifying multilevel predictors of behavioral outcomes like park use: A comparison of conditional and marginal modeling approaches
Source: PLoS One. 2024 Apr 16;19(4):e0301549. doi: 10.1371/journal.pone.0301549 (PMC11020402; doi:10.1371/journal.pone.0301549)
Supplement: S1 File — (DOCX) [file pone.0301549.s002.docx]

**Supplemental File 1.** **Sample code for conditional models, marginal models, and ten fold cross validation.**

**************************************************************;

**************************************************************;

****************Regression/modelling**************************;

**************************************************************;

**************************************************************;

************Full, conditional model**************************;

**proc** **glimmix** data = data;

class Parkuse parkID pptID gender education (ref="1") race physical_activity(ref="1") MHHI (ref="1")

pop_density(ref="3");

model Parkuse (ref="0")=

**ppt level**; age gender education race PA_tertimp

**park level**; ParkQuality distance acres_perpark

**neighborhood level**; number_of_parks MHHI pop_density Percent_White Percent_over65

walkability/ dist=binary link=logit or ddfm=satterth solution STDCOEF;

random intercept / subject = GEOID_BG type = vc;

random intercept / subject = ID5 (GEOID_BG) type = vc;

run;

*****Reduced,conditional model***********;

**proc** **glimmix** data = data;

class Parkuse parkID pptID education (ref="1") race physical_activity(ref="1") MHHI (ref="1")

pop_density(ref="3");

model Parkuse (ref="0")=

**ppt level**; education race

**park level**; ParkQuality distance park_size

**neighborhood level**; Percent_over65

/dist=binary link=logit or ddfm=satterth solution STDCOEF;

random intercept / subject = GEOID_BG type = vc;

random intercept / subject = ID5 (GEOID_BG) type = vc;

run;

************Full, marginal model**************************;

**proc** **gee** data = data;

class Parkuse parkID ID5 gender education (ref="1") race physical_activity(ref="1") MHHI (ref="1")

pop_density(ref="3");

model Parkuse (ref="0")=

**ppt level**; age gender education race physical_activity

**park level**; ParkQuality distance acres_perpark

**neighborhood level**; number_of_parks MHHI pop_density Percent_White Percent_over65

walkability/ dist=bin link=logit type3 scale=deviance;

repeated subject = ID5 (GEOID_BG)/type=ind corrw;

run;

**proc** **gee** data = data;

class Parkuse parkID pptID education (ref="1") race MHHI (ref="1");

model Parkuse (ref="0")=

**ppt level**; education race

**park level**; ParkQuality distance

**neighborhood level**; Percent_over65

/dist=bin link=logit type3 scale=deviance;

repeated subject = ID5 (GEOID_BG)/type=ind corrw;

run;

**************************************************************;

**************************************************************;

**************** Cross validation **************************;

**************************************************************;

**************************************************************;

*This is for K=10 fold cross-validation. If you want to do less/more change the PROC SURVEYSELECT below.

*for example, if you wanted to do 5 you would set "samprate = 4/5" and "reps = 5". Everything else would stay the same.

**************************************************************;

**************************************************************;

***** Conditional model – fixed and random effects************;

**************************************************************;

**************************************************************;

*Generate the cross validation sample;

**proc** **surveyselect** data= data out=cv_data seed=**231258**

samprate=**90** outall reps=**10**;

**run**;

/* this should create a dataset of n*10 values, each observation should be missing once.*/

**data** cv_data;

set cv_data;

if selected then new_Parkuse = Parkuse;

**run**;

/* get predicted values for the missing new_Parkuse in each replicate */

ods output ParameterEstimates=ParamEst;

**proc** **glimmix** data = cv_data;

by replicate;

class Parkuse parkID pptID gender education (ref="1") race physical_activity(ref="1") MHHI (ref="1")

pop_density(ref="3");

model Parkuse (ref="0")=

**ppt level**; age gender education race PA_tertimp

**park level**; ParkQuality distance acres_perpark

**neighborhood level**; number_of_parks MHHI pop_density Percent_White Percent_over65

walkability/ dist=binary link=logit or ddfm=satterth solution STDCOEF;

random intercept / subject = GEOID_BG type = vc;

random intercept / subject = ID5 (GEOID_BG) type = vc;

output out=out1(where=(new_Parkuse =**.**)) predicted=y_hat; ************predicted statement specifies whether random effects included*************;

run;

/* summarise the results of the cross-validations */

**data** out2;

set out1;

Y_hat2 = (exp(y_hat))/(**1**+(exp(y_hat)));

D = parkuse- y_hat2;

absd=abs(d);

**run**;

**proc** **summary** data=out2;

var d absd;

output out=out3 std(d)=rmse mean(absd)=mae;

**run**;

/* models with a small rmse and mae are better at prediction */

**************************************************************;

**************************************************************;

***** Conditional model – only fixed effects *****************;

**************************************************************;

**************************************************************;

*Generate the cross validation sample;

**proc** **surveyselect** data= data out=cv_data seed=**231258**

samprate=**90** outall reps=**10**;

**run**;

/* this should create a dataset of n*10 values, each observation should be missing once.*/

**data** cv_data;

set cv_data;

if selected then new_Parkuse = Parkuse;

**run**;

/* get predicted values for the missing new_Parkuse in each replicate */

ods output ParameterEstimates=ParamEst;

**proc** **glimmix** data = cv_data;

by replicate;

class Parkuse parkID pptID gender education (ref="1") race physical_activity(ref="1") MHHI (ref="1")

pop_density(ref="3");

model Parkuse (ref="0")=

**ppt level**; age gender education race PA_tertimp

**park level**; ParkQuality distance acres_perpark

**neighborhood level**; number_of_parks MHHI pop_density Percent_White Percent_over65

walkability/ dist=binary link=logit or ddfm=satterth solution STDCOEF;

random intercept / subject = GEOID_BG type = vc;

random intercept / subject = ID5 (GEOID_BG) type = vc;

output out=out1(where=(new_Parkuse =**.**)) pred(NOBLUP ILINK)=y_hat; ************predicted statement specifies whether random effects included – here has “NOBLUP ILINK” to remove random effects*************;

run;

run;

/* summarise the results of the cross-validations */

**data** out2;

set out1;

Y_hat2 = (exp(y_hat))/(**1**+(exp(y_hat)));

D = parkuse- y_hat2;

absd=abs(d);

**run**;

**proc** **summary** data=out2;

var d absd;

output out=out3 std(d)=rmse mean(absd)=mae;

**run**;

/* models with a small rmse and mae are better at prediction */

************************************;

************************************;

******** Marginal model ************;

************************************;

************************************;

*This is for K=10 fold cross-validation. If you want to do less/more change the PROC SURVEYSELECT below.

*for example, if you wanted to do 5 you would set "samprate = 4/5" and "reps = 5". Everything else would stay the same.

*Generate the cross validation sample;

**proc** **surveyselect** data= data out=cv_data seed=**231258**

samprate=**90** outall reps=**10**;

**run**;

/* this should create a dataset of n*10 values, each observation should be missing once.*/

**data** cv_data;

set cv_data;

if selected then new_Parkuse = Parkuse;

**run**;

/* get predicted values for the missing new_Parkuse in each replicate */

ods output ParameterEstimates=ParamEst;

**proc** **gee** data = cv_data;

by replicate;

class Parkuse parkID ID5 gender education (ref="1") race physical_activity(ref="1") MHHI (ref="1")

pop_density(ref="3");

model Parkuse (ref="0")=

**ppt level**; age gender education race physical_activity

**park level**; ParkQuality distance acres_perpark

**neighborhood level**; number_of_parks MHHI pop_density Percent_White Percent_over65

walkability/ dist=bin link=logit type3 scale=deviance;

repeated subject = ID5 (GEOID_BG)/type=ind corrw;

run;

output out=out1(where=(new_Parkuse =**.**)) predicted=y_hat;

run;

/* summarise the results of the cross-validations */

**data** out2;

set out1;

Y_hat2 = (exp(y_hat))/(**1**+(exp(y_hat)));

D = parkuse- y_hat2;

absd=abs(d);

**run**;

**proc** **summary** data=out2;

var d absd;

output out=out3 std(d)=rmse mean(absd)=mae;

**run**;

/* models with a small rmse and mae are better at prediction */
