# Supplementary figures and images for: Identifying multilevel predictors of behavioral outcomes like park use: A comparison of conditional and marginal modeling approaches
Source: PLoS One. 2024 Apr 16;19(4):e0301549. doi: 10.1371/journal.pone.0301549 (PMC11020402; doi:10.1371/journal.pone.0301549)

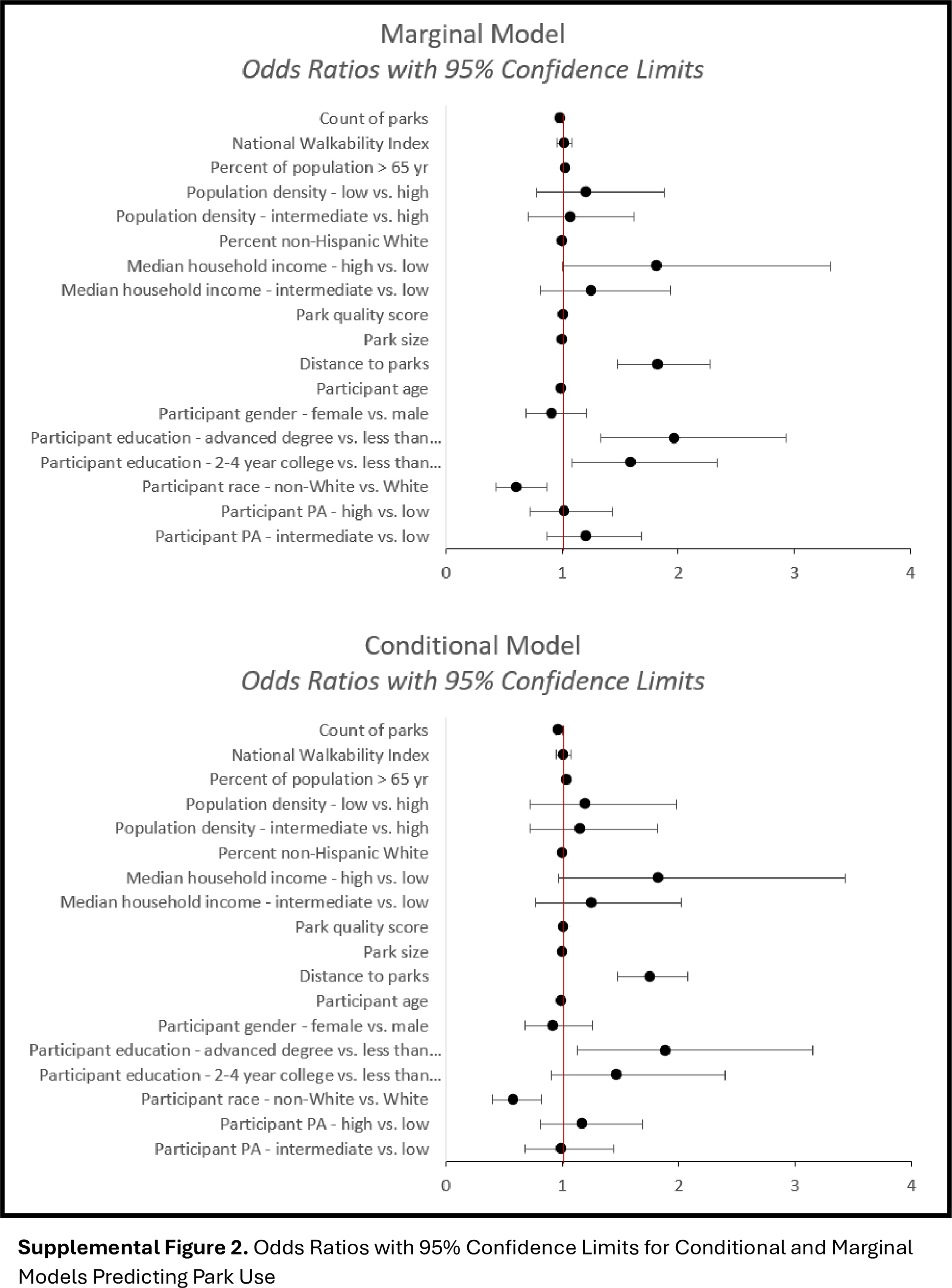

Supplement: S1 Fig — (TIF) [file pone.0301549.s004.tif]
