# Supplementary material for: Identifying multilevel predictors of behavioral outcomes like park use: A comparison of conditional and marginal modeling approaches
Source: PLoS One. 2024 Apr 16;19(4):e0301549. doi: 10.1371/journal.pone.0301549 (PMC11020402; doi:10.1371/journal.pone.0301549)
Supplement: Attachment — Submitted filename: ID PONE-D-23-37844 Reviewer.doc [file pone.0301549.s005.doc]

**Comment on Manuscript**

Manuscript Number: **PONE-D-23-37844**

Full Title: **Identifying Multilevel Predictors of Behavioral Outcomes Like Park Use: A Comparison of Conditional and Marginal Modeling Approaches**

*Submitted to* ***Plos One*** *(SCIE, IF = 3.7)*

The authors have studied the marginal and the conditional approaches for identifying individual, park, and neighborhood park use predictors. There are some collected results in this study however there are some drawbacks that the authors should address them to improve this study.

1. In the introduction, the main contribution of this study must be provided to outline the prioritization of this comparative work.
2. The proposed models about multilevel predictors of behavioral outcomes must be provided in this study.
3. The description of dataset must be indicated and presented through the distribution areas as well as the specific database.
4. The collected results must be presented through the distributive data figures.
5. The comparative methods must be implemented in the discussion.
6. The novelty points of this study must be indicated clearly in the conclusion part.
